# Association between IL-17 and IgA in the joints of patients with inflammatory arthropathies

**DOI:** 10.1186/s12865-017-0189-9

**Published:** 2017-02-06

**Authors:** Ricardo Javier Eliçabe, Juan Eduardo Silva, Mabel Noemí Dave, María Gabriela Lacoste, Héctor Tamashiro, Rodrigo Blas, Alicia Munarriz, Gabriel Adrián Rabinovich, María Silvia Di Genaro

**Affiliations:** 1 Division of Immunology, Faculty of Chemistry, Biochemistry and Pharmacy, National University of San Luis, Ejército de los Andes 950, San Luis, Argentina; 2 Laboratory of Immunopathology, Multidisciplinary Institute of Biological Investigations - San Luis (IMIBIO-SL), National Council of Scientific and Technical Investigations (CONICET), Ejército de los Andes 950, San Luis, 5700 Argentina; 3 Private Medical Clinic Bolivar, Bolivar 1277, San Luis, 5700 Argentina; 4 Medical Clinic Medici, Ayacucho 1118, San Luis, 5700 Argentina; 5 CENYR Center, Ituzaingo 1074, San Luis, 5700 Argentina; 6 Laboratory of Immunopathology, Institute of Biology and Experimental Medicine (IBYME), CONICET, Vuelta de Obligado 2490, Buenos Aires, C1428A Argentina; 7 Faculty of Exact and Natural Sciences, University of Buenos Aires, Intendente Güiraldes 2160, Buenos Aires, C1428EGA Argentina

**Keywords:** Interleukin-17, Immunoglobulin A, Synovial fluid, Transforming growth factor, B-cell activating factor of the TNF family

## Abstract

**Background:**

Hyperactive secretion and pathogenic effects of interleukin (IL)-17 and IgA have been detected in different arthropathies. Recent evidence has revealed that T_H_17 cytokines regulate mucosal IgA secretion. However, it is unknown whether and how IL-17 mediates synovial IgA production. Here we aim to investigate the connection of synovial IL-17 with IgA production in the joint.

In this study we included synovial fluids (SF) from patients with rheumatoid arthritis (RA; *n* = 66), spondyloarthritis (SpA; *n* = 18) and osteoarthritis (OA; *n* = 36). The levels of IL-17, IL-6, transforming growth factor (TGF)-β_1_, B-cell-activating factor of the TNF family (BAFF) and anti-lipopolyssacharide (LPS) immunoglobulin (Ig)A were investigated by enzyme-linked immunosorbent assay (ELISA). Total IgA was measured by radial immunodiffusion assay. Synovial fluid-derived mononuclear cells (SFMC) were stimulated with bacterial antigens or SF-conditioned media, and cytokines and IgA were analyzed in the supernatants.

**Results:**

IL-17, IL-6 and TGF-β_1_ were increased in SF from both RA and SpA compared with OA patients. Concentration of IL-17 correlated with the disease activity score (DAS)-28, IL-6 and anti-LPS IgA levels. Bacterial-stimulated SFMCs from RA and SpA patients secreted higher IL-17 than vehicle-stimulated SFMCs. Conditioned media with SF containing IL-17 induced anti-LPS IgA production by SFMCs which was independent of IL-6 activity. Concentrations of synovial TGF-β_1_ and BAFF correlated with anti-LPS and total IgA levels, respectively. Blockade of IL-17 decreased the production of TGF-β_1_ and anti-LPS IgA by SF-stimulated SFMCs.

**Conclusions:**

This study reports a connection between IL-17 and IgA secretion in the joint. In addition, it demonstrates that enterobacterial antigens trigger synovial IL-17 production, and that TGF-β_1_ and BAFF may mediate the effect of IL-17 on IgA production. This circuit may contribute to the pathogenesis of inflammatory joint diseases.

## Background

Mucosal surfaces serve as a protective barrier against most pathogens. These surfaces are protected by a first-line defense mediated by immunoglobulin A (IgA) [1]. Moreover, it is also known that T helper (T_H_)17 cells are more abundantly present at the mucosal surface of the intestine, compared with other T-cell subsets [2]. Accumulating evidence has demonstrated that T_H_17 cells contribute to intestinal homeostasis by regulating intestinal IgA secretion supporting a link between intestinal T-cell function and IgA production [3, 4]. Less is known about the potential role of T_H_17 cells for IgA induction in the joint, though chronic activation of T_H_17 can cause arthropathies [5, 6] and hyperactive IgA synthesis occurs in many types of inflammatory joint diseases [7–10].

In spite of the long-standing assumption that infectious agents and their products may influence the development of osteoarticular diseases [11, 12], our understanding of the intricate connections linking bacterial components with inflammatory arthropathies is still limited. Rheumatoid arthritis (RA), one of the most common autoimmune osteoarticular diseases, is characterized by synovial inflammation and hyperplasia, autoantibody production, cartilage and bone destruction and systemic disorders mainly driven by pro-inflammatory cytokines and matrix-degrading enzymes [13]. Infectious agents and their products have been largely linked with RA although the precise mechanisms implicated in this complex relationship is not fully understood [11, 12, 14]. Elucidation of the pathogenic mechanisms that perpetuate RA will open the possibility of better disease management and rational control of immune dysregulation [13]. Spondyloarthritis (SpA) is the second most prevalent form of chronic inflammatory arthritis (prevalence 0.5-1.5%) that mainly affects young adults [15]. SpA has been traditionally subdivided into subtypes including ankylosing spondylitis (AS), psoriatic arthritis (PsA), reactive arthritis (ReA), arthritis/spondylitis associated with inflammatory bowel disease (IBD), and undifferentiated SpA (uSpA) [16]. However, evidence suggests that SpA is a single disease with a heterogeneous phenotype [17]. Although the etiology of SpA remains obscure, it has demonstrated a strong association with environmental factors including pathogenic intestinal microbes [18]. However, whether enterobacterial antigens trigger synovial IL-17 in RA and SpA is still uncertain.

It is known that transforming growth factor (TGF)-β_1_ has a unique role in driving IgA isotype switching [19], and is also critical for T_H_17-cell differentiation [20]. It is also known that IL-6, a major factor for the differentiation of naïve CD4 T cells into Th17 cells [21], plays a key role in B-cell proliferation and antibody secretion [22]. However, there is no evidence indicating that TGF-β_1_ or IL-6 together with IL-17 have a role for IgA generation in joint. Ultimately, B- cell-activating factor of the TNF family (BAFF) has been recognized as a cytokine that induces IgA class switching by activating B cells [23]. Interestingly, IL-17 has also been recently shown to synergize with BAFF to increase the frequency of autoantibodies, and high BAFF levels have been considered a measurement of B-cell dysfunction in autoimmune diseases [24].

In the present study, we aimed to examine the role of IL-17 in IgA production in the joint. We used synovial fluids (SF) containing different cytokines rather recombinant cytokines in order to recreate the pathophysiologic microenvironment of the joints during ongoing inflammatory diseases. These findings identify pathways by which IL-17 mediates induction of synovial IgA responses during inflammatory arthropathies.

## Methods

### Patients

Synovial fluids (SF) were obtained from 66 RA, 18 SpA and 36 osteoarthritis (OA) patients from San Luis, Argentina. Since OA is not associated with infections, these patients were considered a control group. The RA and SpA patients met the American College of Rheumatology (ACR) [25] and the European Spondyloarthopathy Study Group (ESSG) criteria [26], respectively. Table 1 shows the demographic features of patients. To enroll in the study, patients in all groups required knee effusion of SF. Age, sex and disease duration were recorded (Table 1). Fifty six RA patients were classified as late stage disease according to the ACR criteria [25]. Eight patients with early SpA were categorized as having disease of less than 6 month-duration (Table 1). The 28-joints disease activity score (DAS28) was used as a measure of disease activity in RA [27]. The medium DAS28 of the RA patients was 4.64 ± 1.6 (range: 2.42–7.1). The 95%, 44.5% and 21.4% of RA, SpA and OA patients, respectively were positive for C-reactive protein (CRP). The SpA group included patients with AS (1), PsA (5), ReA (3), IBD (1) and uSpA (8). The current medications were recorded and included: nonsteroidal anti-inflammatory drugs in 39 patients with RA and 10 with SpA; methotrexate in 40 patients with RA and 11 with SpA; leflunomide in 16 patients with RA; hydroxychloroquine in 12 patients with RA; sulfasalazine in 6 patients with RA and 8 with SpA; anti-TNF in 6 patients with RA and 1 with SpA; prednisone in 24 patients with RA. Combined medication was used in the majority of the patients. Ten patients with RA had no treatment at the moment of study enrolment since SF drainage was required in their first visit to the rheumatologist.

**Table 1:** Demographic characteristics of the patients

Disease	Patient number	M:F	Medium age, years (range)	Medium duration of disease, years (range)
RA	66	12:54	55 (20–74)	8 (3 months-30 years) 85% chronic phase (>1 year)
SpA	18	14:4	33 (12–59)	4 (2 months-17 years) 55% chronic phase (>6 months)
OA	36	11:25	61 (42–83)	-

*RA* rheumatoidarthritis, *SpA* spondyloarthritis, *OA* osteoarthritis, *M:F* male:female

### Ethical aspects

The study was approved by the Ethics Committee of the National University of Cuyo, Mendoza, Argentina. Informed consent was obtained from all patients included in the study. The principles of Helsinki Declaration 1975/83 were followed.

### Synovial fluid preparation

SF was aspirated from knee joints and mixed immediately with 50 IU/ml heparin. The volumes of aspired fluids were 5–30 ml. SF of RA and SpA patients were classified as inflammatory since all of them had more than 2000 cells/mm^3^; in contrast, OA patients had non-inflammatory SF (less than 2000 cells/mm^3^) [28, 29]. For cytokine and antibody analysis, samples were centrifuged at 250 x xg for 10 min and the supernatants were stored at -20 °C.

### Cytokine determination by enzyme-linked immunosorbent assay (ELISA)

IL-17, TGF-β_1_ and IL-6 were determined in SF using commercial capture ELISA kits (eBioscience, San Diego, CA, USA), according to the manufacturer’s instructions. The limits of detection for the above-mentioned assays were 4 pg/ml for IL-17, 2 pg/ml for IL-6 and 8 pg/ml for TGF-β_1_. The SF with levels of these cytokines higher than the limit of detection was considered positive in the frequency analysis. BAFF was determined by ELISA kit (Antigenic America, Huntington Station, NY, USA) which was kindly provided by Dr. Adriana Gruppi (National University of Córdoba, Argentina).

### Enterobacterial antigen preparations

*Yersinia enterocolitica* O:8, strain WA-314 (kindly provided by Dr Kapperud, Department of Bacteriology, Oslo, Norway) was used for heat killed *Yersinia* (HKY) preparation, which consisted in a twice autoclaved bacterial suspension (1 × 10^10^ bacteria/ml). The absence of bacterial growth in HKY was tested by plating on Mueller-Hinton agar and incubation at 26 °C for 48 h. Lipopolysaccharide (LPS) was obtained as previously described [30].

### Synovial anti-enterobacterial IgA and total IgA

Multiwell plates were coated with 100 μl per well of 10 μg/ml LPS in 0.15 M phosphate-buffered saline (PBS) pH 7.2 at 4 °C overnight. After incubation with 1:50 diluted SF, bound antibodies were demonstrated by reaction with goat anti-human IgA and peroxidase-conjugated rabbit anti-goat IgG (Sigma, St. Louis, MO, USA) followed by the addition of the enzyme substrate (H_2_O_2_) and chromogen O-phenylendiamine (Sigma). Optical density (OD) was measured at 490 nm in an ELISA reader (Bio-Rad, Hercules, CA, USA). Total IgA levels in SF were determined by radial immunodiffusion assay (Diffu-Plate kit, Biocientífica, Buenos Aires, Argentina)

### Assessment of IL-17, TGF-β1 and anti-LPS IgA in stimulated mononuclear cells from SF

SF mononuclear cells (SFMC) were obtained using Ficoll-Hypaque (Histopaque 1077, Sigma). Cells (2 × 10^6^ cells/well) were cultured in RPMI 1640 medium (Hyclone, Logan, UT, USA) with 10% fetal bovine serum (FBS) (Sigma), and stimulated with 10^7^ or 10^8^ bacteria/ml of HKY and incubated at 37 °C in 5% CO_2_. Supernatants were collected after 72 h for determination of IL-17 using a commercial ELISA kit (eBioscience). In addition, SFMC were incubated for 96 h with media conditioned with different dilutions of SF containing IL-17; then, TGF-β_1_ or anti-LPS IgA were measured by ELISA. To analyze the role of IL-6 in this effect, cells were incubated in vitro with pharmacologically relevant concentrations of the anti-IL-6 receptor antagonist tocilizumab (TCZ) (200 μg/ml, Roche Pharma, Grenzach-Wyhlen, Germany) [31]. To analyze the effect of synovial IL-17, the cells were stimulated with SF in presence of the anti-IL-17 (100 μg/ml, Secukinumab, Novartis Argentina SA, Buenos Aires, Argentina).

### Statistical analysis

Differences in the amounts of cytokines or IgA in RA, SpA and OA patients were compared by using one-way analysis of variance (ANOVA) followed by Tukey’s multiple comparison test. Two variables were compared by unpaired Student´s *t* test. Differences in the frequencies of SF with detectable level of cytokine (positive SF) in each group of patients were analyzed by Fisher´s exact test. Correlations between two variables were examined by Spearman’s analysis. A *P* value less than 0.05 was considered as statistically significant. All analyses were performed using GraphPad Prism 5 software (GraphPad Software, San Diego, CA, USA).

## Results

### Synovial IL-17, IL-6 and TGF-β_1_ production in patients with RA and SpA

**Fig. 1:**
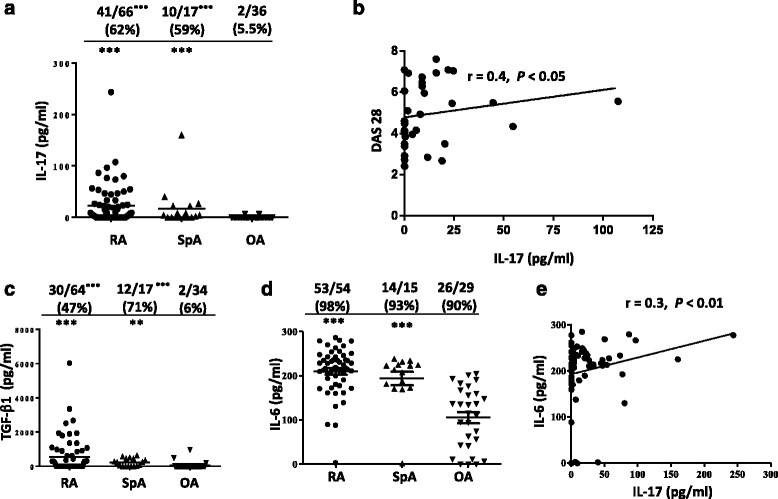
Levels of IL-17, IL-6 and TGF-β_1_ in synovial fluids from patients with arthritis. Concentrations of cytokines and frequencies of patients with detectable concentrations of IL-17 (**a**), TGF-β_1_ (**c**), and IL-6 (**d**) in synovial fluids (SF) of patients with rheumatoid arthritis (RA), spondyloarthritis (SpA) and osteoarthritis (OA). Bars show the mean. **P < 0.01, ***P < 0.0001compared with OA. **b** Correlation between the IL-17 concentration and DAS28 in SF from patients with RA. **e** Correlation between the concentrations of IL-6 and IL-17 in SF of RA and SpA patients. The correlations were assessed by the Spearman’s rank correlation

Since IL-17 has been associated with the pathogenesis of RA and SpA [32, 33], we first studied this cytokine in SF from RA and SpA in comparison with OA patients. Next, we analyzed the cytokines TGF-β_1_ and IL-6 that in combination are required for T_H_17 development [21]. The frequency of SF with detectable levels of each cytokine was also compared. We found a higher number of patients with detectable synovial IL-17 in RA and SpA compared to OA (62% and 59%, respectively versus 5.5%) (*P* < 0.0001 compared with OA for both comparisons). Of note, the IL-17 median concentration was significantly higher in SF from RA or SpA compared to OA patients (*P* < 0.0001) (Fig. 1a). We next examined whether an association exists between the levels of IL-17 and disease activity in RA. We found that synovial production of IL-17 correlated with RA disease activity as measured by DAS28 (*r* = 0.4, *P* < 0.05) (Fig. 1b). The determination of TGF-β_1_ revealed similar differences as those observed for IL-17 as higher percentage of RA or SpA patients had detectable levels of TGF-β_1_ in their SF (*P* < 0.0001 compared with OA for both comparisons) and the median values of this cytokine were higher in SF from both RA and SpA patients (*P* < 0.0001 RA vs OA, and *P* < 0.01 SpA vs OA) (Fig. 1c). Furthermore, a high percentage of RA, SpA and OA patients had IL-6 in their SF (98%, 93% and 90%, respectively), while the median concentration of this cytokine was significantly elevated in RA and SpA patients compared with OA patients (*P* < 0.0001 for both comparisons) (Fig. 1d). Remarkably, when we analyzed the relationships between the different cytokines, we observed a positive correlation between IL-6 and IL-17 in SF from RA and SpA patients (*r* = 0.3; *P* < 0.01) (Fig. 1e).

### Secretion of IL-17 by SFMCs from patients with RA and SpA after stimulation with enterobacterial antigens and anti-enterobacterial IgA response

**Fig. 2:**
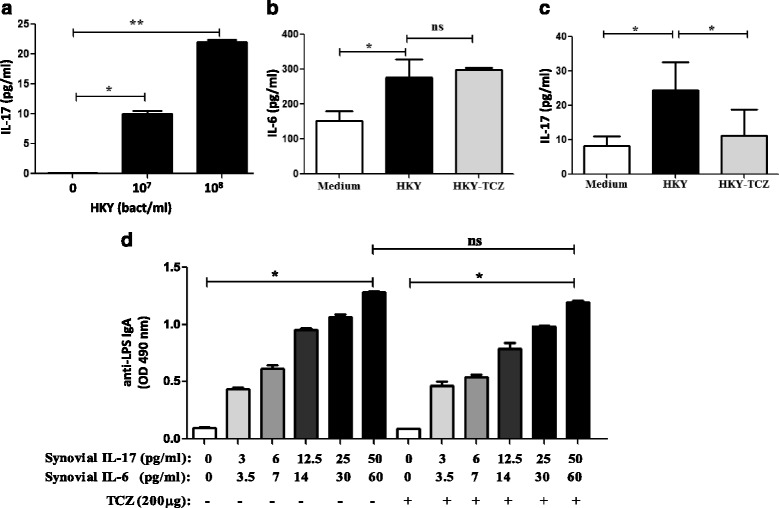
Association between IL-17 production and IgA anti-enterobacterial antigens. **a** Dose-dependent IL-17 production in culture supernatants of SF mononuclear cells (SFMC) (2 × 10^6^ cells/well) after 72 h stimulation with 10^7^ or 10^8^ bacteria/ml of heat-killed *Yersinia* (HKY). Concentrations of IL-6 (**b**) and IL-17 (**c**) in culture supernatants of SFMC (2 × 10^6^ cells/well) following 72 h stimulation with 10^8^ HKY with or without tocilizumab (TZC). **d** Anti-LPS IgA in culture supernatants of SFMC (2 × 10^6^ cells/well) following 96 h of stimulation with media conditioned with SF containing different concentrations of IL-17 and IL-6 with or without tocilizumab (TZC). Data are expressed as mean ± SD. *P < 0.05, **P < 0.01

We investigated a possible causative link between bacterial antigens and synovial IL-17 production. Therefore, we evaluated the effects of stimulating with HKY in SFMC obtained from RA and SpA patients. We found that SFMC from RA and SpA patients secreted significantly higher amounts of IL-17 following stimulation, and this effect was found to be dose-dependent when different HKY concentrations were used (Fig. 2a). Consistent with our previous findings demonstrating a correlation between IL-17 and IL-6, we found that IL-6 was also induced by HKY-stimulation of SFMC (Fig. 2b). In presence of TCZ, an inhibitor of IL-6 function, although IL-6 was secreted (Fig. 2b), IL-17 production by HKY-stimulated SFMC significantly decreased (Fig. 2c). These results suggest that IL-6 mediated IL-17 secretion by enterobacterial antigen in SFMC. To study the influence of IL-17 on the induction of IgA in the joint, we next analyzed anti-LPS IgA in supernatants of SFMC stimulated with media conditioned with different dilutions of SF with high IL-17 and IL-6 concentrations. We found a dose-dependent induction of anti-LPS IgA by IL-17 which could not be inhibited by TCZ (Fig. 2d). Our data suggest that production of IgA specific to enterobacterial antigens is associated with IL-17 levels and that IL-6 is not essential for IL-17-induced IgA responses.

### Interaction of IL-17, TGF-β_1_ and BAFF in synovial IgA production

**Fig. 3:**
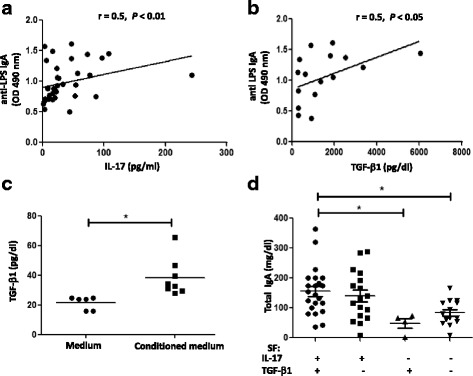
Interaction of IL-17 and TGF-β_1_ for synovial IgA production. Correlation of IL-17 (**a**) or TGF-β_1_ (**b**) levels with anti-LPS IgA response in SF from patients with RA and SpA (Spearman’s rank correlation). **c** TGF-β_1_ production in culture supernatants of SF mononuclear cells (SFMCs) (2 × 10^6^ cells/well) following 96 h of stimulation with medium conditioned with different SF containing IL-17 (20 pg/ml). **d** Total IgA concentrations in SF with IL-17 and TGF-β_1_ in detectable (+) or undetectable (-) levels. Bars show the mean.*P < 0.05

**Fig. 4:**
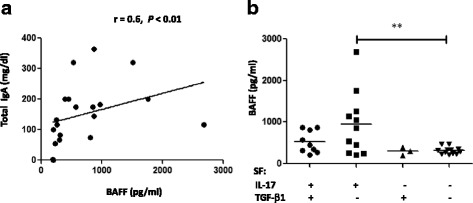
IL-17 and BAFF association for synovial IgA production. **a** Correlation between BAFF and total IgA concentrations in synovial fluids (SF) from the patients (Spearman’s rank correlation). **b** BAFF concentrations in SF with IL-17 and TGF-β_1_ in detectable (+) or undetectable (-) levels. Bars show the mean.**P < 0.01

**Fig. 5:**
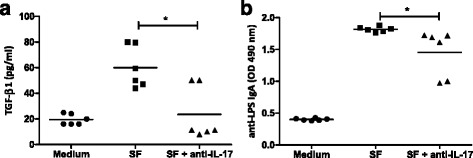
Blockade of IL-17 decreases the induction of TGF-β_1_ and anti-LPS IgA in SF-stimulated SFMCs. TGF-β_1_ (**a**) and anti-LPS IgA (**b**) in supernatants of SF mononuclear cells (SFMCs) (2 × 10^6^ cells/well) following stimulation with media conditioned with synovial fluid (SF) containing IL-17 in presence of anti-IL-17.*P < 0.05. Bars show the mean

Since SF recapitulates the microenvironment of joints, we measured anti-enterobacterial IgA in the SF from the patients to correlate the levels of this immunoglobulin with IL-17 concentration. Our results revealed a significant correlation between IL-17 production and anti-LPS IgA antibodies in SF from RA and SpA patients (*r* = 0.5; *P* < 0.01) (Fig. 3a). This correlation raised the possibility that IL-17 may contribute to LPS-specific IgA responses. TGF-β_1_ has been shown to have a direct role on class switching to IgA [19, 34, 35], and we also demonstrated a positive correlation between LPS-specific IgA response and TGF-β_1_ levels in SF from RA and SpA patients (*r* = 0.5; *P* < 0.05) (Fig. 3b). Therefore, we explored a possible indirect mechanism by which IL-17 could influence IgA production through TGF-β_1_. We detected TGF-β_1_ induction in SFMCs stimulated with medium conditioned with different SF containing IL-17 (20 pg/ml) and undectable TGF-β_1_ levels (Fig. 3c). Finally, we found that SF with detectable levels of IL-17 and TGF-β_1_ had significantly higher levels of total IgA compared with those with undetectable levels of both cytokines (Fig. 3d). Remarkable, SF containing TGF-β_1_ and lacking IL-17 had very low concentration of IgA. However, SF containing IL-17 but lacking TGF-β_1_ showed high concentrations of IgA (Fig. 3d). In addition, we analyzed BAFF as this cytokine has been associated with IL-17 in antibody responses [24]. As expected, we observed correlation between BAFF and IgA concentrations in the SF from patients (Fig. 4a). Interestingly, we found significantly higher BAFF levels in SF that were IL-17-positive but TGF-β_1_-negative^-^ (Fig. 4b). In addition, anti-IL-17 decreased TGF-β_1_ (Fig. 5a) and anti-LPS IgA (Fig. 5b) in the supernatants of SFMC stimulated with SF, supporting the role of IL-17 contained in the SF on the production of TGF-β_1_ and anti-LPS IgA by IL-17 sensitive cells in SFCM. In summary, these data suggest that in the joint microenvironment IL-17 functions to induce synovial IgA in association with TGF-β_1_ and BAFF.

## Discussion

The role of IL-17 in the induction of IgA in the joint is poorly understood. Most of the available information on the association between Th17 and IgA comes from studies of intestinal immunity. In this regard, recent studies have demonstrated that T_H_17 cells are the T helper subset responsible for promoting the B cell switch toward the production of high-affinity T cell-dependent IgA responses [36]. Moreover, microbial-specific Th17 cells contribute to intestinal homeostasis by regulating intestinal pIgR expression and IgA secretion [4]. In the present work we focused on IL-17-mediated induction of IgA production in the joint.

First, we detected elevated IL-17 concentrations in SF from both RA and SpA patients compared with control OA patients. These observations are in accordance with previous studies in RA and in SpA [6, 32, 33] patients. Furthermore, differences in SF concentrations of IL-6 and TGF-β_1_ were similar to those found for IL-17, and positive correlation was also observed between SF concentrations of IL-17 and IL-6. These results are consistent with the role of IL-6 and TGF-β_1_ in the differentiation of IL-17-producing CD4^+^ T cells [20, 21, 37] and support the involvement of IL-17 in the pathogenesis of the inflammatory arthritis (RA and SpA) but not in OA. Noteworthy, we detected a positive association between IL-17 and disease activity evaluated by DAS28 in RA. In accordance, in other study, Th17 cells positively correlated with both CRP levels and DAS28 in RA patients [6]. Although this correlation was more difficult to assess in SpA, accumulating evidence firmly implicates IL-17 in the pathogenesis of SpA [33, 38].

In spite of considerable progress in the elucidation of the relationship between bacterial infection and rheumatologic disorders [11, 12, 14], the precise mechanism by which bacterial enteroantigens modulate the local inflammatory microenvironment and influence clinical course of inflammatory arthropathies are poorly understood. Here, we found that stimulation with *Yersinia* antigens promotes robust IL-17 production by SFMC of RA and SpA patients. This induction was IL-6-dependent in line with the well-established role of IL-6 as a potent inducer of T_H_17 differentiation [21, 37], and the major role of IL-6 in the pathophysiology of arthritis [39]. Additionally, we demonstrated an IL-17-IgA link in the joint since SFMC secreted anti-LPS IgA in response to stimulation with medium conditioned with SF containing different IL-17 concentrations (Fig. 2d). In line with this finding, a relationship between T_H_17 and B-cell differentiation has been identified [40]. In addition, it has been recently reported that lung IgA response is dependent on T_H_17 cells since depletion of IL-17 ablates IgA responses in the lung [41]. Moreover, IL-17 has been involved simultaneously in both aggravating intestinal inflammation and promoting the development of rapidly progressive IgA nephropathy in patients with Crohn’s disease [42]. These findings demonstrate a connection between bacterial stimulation, IL-17 and promotion of local IgA response in arthropathies. We found that IL-6 was not essential for IL-17 effects. In line, IL-6 was not required for IgA^+^ B cell development or specific mucosal IgA responses in other in vivo systems [43, 44].

We demonstrated that SF with elevated IL-17 and TGF-β_1_ levels had higher anti-LPS IgA levels reinforcing T_H_17-IgA connection in arthritis. TGF-β_1_ is a direct regulator of class switching to IgA [19, 45, 46]. Therefore, to investigate whether IL-17 works indirectly for helping the synovial IgA response, we analyzed the relationship between IL-17 and TGF-β_1_. Our data indicated that medium conditioned with SF containing IL-17 induced TGF-β_1_ production by SFMC. Accordingly, the SF with both cytokines showed the higher levels of IgA in SF. Interestingly, SF containing TGF-β_1_ but lacking IL-17 showed low IgA concentration suggesting that IL-17 or T_H_17 cytokines may play an essential role for synovial IgA generation. A recent report further demonstrated that T_H_17 cells may convert into T follicular helper (T_FH_) cells in Peyer’s patches and induce intestinal IgA [3]. It has been shown that IL-21 can modulate B cell differentiation by enhancing TGF-β_1_-driven IgA production [46]. Moreover, Cao et al have recently demonstrated that IL-21, produced by both T_H_17 and T_FH_ cells, can augment IgA responses mediated by TGF-β_1_ and retinoic acid in the intestine, and intestinal sources of IL-21 directly induce IgA production [47]. Therefore, the role of IL-21 in IgA generation in the joint will require further investigation. However, it has been also demonstrated that T_H_17 cell cytokines, IL-17 and IL-21 are able to function indirectly to induce IgA production by promoting the expression of BAFF [47]. In line, our data showed that SF with detectable levels of IL-17 but lacking TGF-β_1_ had elevated levels of IgA (Fig. 3d) and BAFF (Fig. 4b). These results indicate alternative promotion of TGF-β_1_ and BAFF by IL-17 for synovial IgA generation. Moreover, blockade of IL-17 functions using a specific monoclonal antibody reduced the induction of TGF-β_1_ and anti-LPS IgA in SF-stimulated SFMCs. We assume that these findings support again the IL-17 effects on the induction of synovial TGF-β_1_ and IgA. Remarkably, IgA concentration has been related with active arthritis [9, 10], and in the last decade it has become clear that IgA is a very potent stimulus to initiate pro-inflammatory cellular processes [48]. Our study demonstrates the association between IL-17 and IgA responses in the microenvironment of the joint.

## Conclusions

Taken together, our results revealed that combined secretion of IL-17, IL-6 and TGF-β_1_ is a hallmark of inflammatory arthropathies. Moreover, our findings sustain the notion that synovial IL-17 is sensitive to bacterial enteroantigens derived from intestinal infections. These data support an association between IL-17 and IgA responses in the joint, and the involvement of TGF-β_1_ and BAFF in the mechanisms of IgA-induction by IL-17.

## Abbreviations

ACR: American College of Rheumatology
ANOVA: analysis of variance
AS: ankylosing spondylitis
DAS28: 28-joints disease activity score
ESSG: the European Spondyloarthopathy Study Group
ELISA﻿﻿: enzyme-linked immunosorbent assay
HKY: heat killed *Yersinia*
IBD: inflammatory bowel disease
IgA: immunoglobulin A
IL: interleukin
LPS: lipopolyssacharide
OA: osteoarthritis
PBS: phosphate-buffered saline
PsA: psoriatic arthritis
RA: rheumatoid arthritis
ReA: reactive arthritis
SF: synovial fluids
SFMCs: synovial fluid-derived mononuclear cells
SpA: spondyloarthritis
TCZ: tocilizumab
TGF-β_1_: transforming growth factor-β_1_
BAFF: B-cell-activating factor of the TNF family
uSpA: undifferentiated SpA
